# 2518 InCHOIR learning lab: A TL1 and workforce development initiative at Mount Sinai

**DOI:** 10.1017/cts.2018.215

**Published:** 2018-11-21

**Authors:** Emma K. T. Benn, Janice L. Gabrilove, Layla Fattah, Emilia Bagiella

## Abstract

OBJECTIVES/SPECIFIC AIMS: Science and clinical practice are widely regarded as being complementary and synergistic. In an effort to enhance the team science, translational research capacity of the TL1 scholars at Icahn School of Medicine at Mount Sinai (ISMMS), the InCHOIR learning lab aims to provide an accessible, workforce-wide lecture series on the fundamental methods and concepts of randomized clinical trials. METHODS/STUDY POPULATION: The InCHOIR learning lab is a monthly 1 hour lecture series delivered by a range of expert clinical and translational researchers, followed by a 1 hour “Meet the Expert” session. The InCHOIR lecture series has covered a wide range of topics including, but not limited to: Decision Models; Race and Causal Inference; Innovative Strategies for Assessing Environmental Health across the Life Course; Statistics for Geneticists and Genetics for Statisticians; and From the Lab to Translation to Policy—The Neuroscience of Addiction. The “Meet the Expert” session offers TL1 predoctoral and postdoctoral scholars and KL2 scholars the opportunity to have intimate, informal discussions with experts about their career trajectories. RESULTS/ANTICIPATED RESULTS: Feedback from participants has been overwhelmingly positive. Participants have gained important insights into key topics relevant to early stage researchers. The “Meet the Expert” sessions have yielded honest and important conversations about crucial topics ranging from finding effective mentors to essential strategies for establishing a work-life balance, to overcoming adversity as underrepresented minorities and women in translational research. DISCUSSION/SIGNIFICANCE OF IMPACT: Attendance at the InCHOIR learning lab is increasing month on month, indicating the perceived need for this learning not just from early stage researchers, but also from students, senior faculty, and research staff more generally. The InChoir series provides added value through the creation of a video library, fostering new collaborations and contributing to the Icahn School of Medicine at Mount Sinai and Graduate Medical Education landscape. Priorities for the program are to increase internal visibility, in order to continue to grow attendance by MSHS students, research staff, nurses, postdoctoral fellows and residents. The program is also exploring how to engage external participation from regional CTSAs and from community advocates actively involved in community-academic research partnerships.

